# Polygenic Nature of Cortical Aging Reveals Cytoskeletal Regulation as a Core Pathway of Resilience

**DOI:** 10.1093/geroni/igaf122.3643

**Published:** 2025-12-31

**Authors:** Ayati Mishra, Nicholas Kim, Nahian Chowdhury, Andrei Irimia

**Affiliations:** University of Southern California, Los Angeles, California, United States; University of Southern California, Los Angeles, California, United States; University of Southern California, Los Angeles, California, United States; University of Southern California, Los Angeles, California, United States

## Abstract

Brain aging is spatially heterogeneous, with some cortical regions more resilient than others. To investigate the genetic basis of this variation, we developed a deep learning architecture to estimate voxel-wise local brain age (LBA) from T1-weighted MRI scans of 41,708 cognitively normal adults (21,983 females) aged 45 to 83 years in the UK Biobank. LBA provides a spatially resolved phenotype of cortical aging, where higher values indicate older-appearing regions. We conducted the first genome-wide association study of LBA and identified 1,212 SNPs significantly associated with region-specific cortical aging. Genome-wide significant SNPs were enriched in biological processes related to cytoskeletal regulation, highlighting it as a key determinant of resilience. Variants in NUAK1, a serine/threonine kinase regulating tau phosphorylation and microtubule stability, were associated with widespread decreases in LBA, suggesting a protective role by promoting cytoskeletal stability and slowing cortical aging. Prior studies link NUAK1 variations to gray matter differences in regions vulnerable to degeneration, and experimental models indicate that its inhibition can reduce tau pathology and mitigates neurodegeneration. Variants in LPAR1, encoding a receptor regulating actin dynamics, neurogenesis, and white matter integrity, were also linked to decreased LBA in parietal and frontal cortices. This aligns with evidence that LPAR1 supports myelination and plasticity through cytoskeletal regulation and prior studies showing loss of LBAR1 leads to demyelinating disease, underscoring its protective role. Altogether, these findings demonstrate that cytoskeletal regulation provides a genetically encoded protective mechanism that helps preserve cortical structure and slow the aging process.

